# The Impact of the Introduction of Innovative REDS Scale for the Evaluation of Central Tunnelled Catheter (CTC) Exit Site on Infection Prevention in Long-Term Haemodialyzed Patients

**DOI:** 10.3389/fsurg.2021.629367

**Published:** 2021-04-09

**Authors:** Tomasz Porazko, Edyta Stasiak, Marian Klinger

**Affiliations:** ^1^Department of Internal Medicine and Nephrology, Institute of Medical Sciences, University of Opole, Opole, Poland; ^2^Department of Nephrology and Dialysis Unit, University Hospital Opole, Opole, Poland; ^3^Doctoral School, Silesian Medical University, Katowice, Poland

**Keywords:** central tunneled catheter, vascular access, chronic kidney disease, hemodialysis, catheter-related infections

## Abstract

Central tunneled catheter (CTC)-related infections are a leading cause of a catheter loss, thus being the source of significant morbidity and mortality. The study aims at evaluating the impact of the implementation of the innovative redness, edema, discharge and tenderness, symptoms (REDS) scale (devised by the authors) for the description of the tunnel condition on the frequency of infection in long-term catheter users. The same cohort of the 40 patients was observed for 4 years altogether: 2 years before and 2 years after REDS application. The results, as well as follow-up evaluation of participants, were compared. The 2-year cumulative incidence of the CTC exit site infection (ESI) dropped significantly (log-rank *p* < 0.001) from 0.89 episode/1,000 catheter days (53.5%, 95% CI [35.9%; 66.2%]) in the period before REDS was used—to 0.26 episode/1,000 catheter days (18.6%, 95% CI [6.1%; 29.4%]) in the time of REDS application. There were also significantly fewer episodes of ESI complicated with catheter-related blood stream infection (CRBSI) requiring the CTC removal (0.6 episode/1,000 catheter days; 18.6%, 95% CI [6.1%; 29.4%] vs. 0.3 episode/1,000 catheter days; 4.7%, 95% CI [0.0; 10.7%]; log-rank *p* = 0.04, in pre-REDS and REDS time, respectively). The REDS scale appears to be a simple, cost-effective tool reducing the frequency of the tunneled CTC exit site infection and associated bloodstream infections.

## Introduction

Central tunneled catheter (CTC) is used in a large part of hemodialysis patients awaiting arteriovenous fistula maturation or as the only possible vascular access option when other ones have been exhausted and/or prevented by comorbidities. Improper use of CTC carries a risk of life-threatening catheter-related bloodstream infections (CRBSI), often complicated with metastatic abscesses, endocarditis, not infrequently with fatal consequence (1–5). Recent studies bring evidence that CTC exit site infection (ESI) may pre-dispose to CRBSI with occurrence in 4 to 20% cases of dialysis line-related sepsis (6, 7). Therefore, a well-designed vascular access care program is of vital importance (8). The purpose of this study was to determine the effect of implementation of the redness, edema, discharge and tenderness, symptoms (REDS) scale exit site evaluation (devised by the authors) on the rate of the catheter infection, as compared with the period when the exit site had been evaluated only at the nursing staff's discretion.

## Materials and Methods

**Figure 1:**
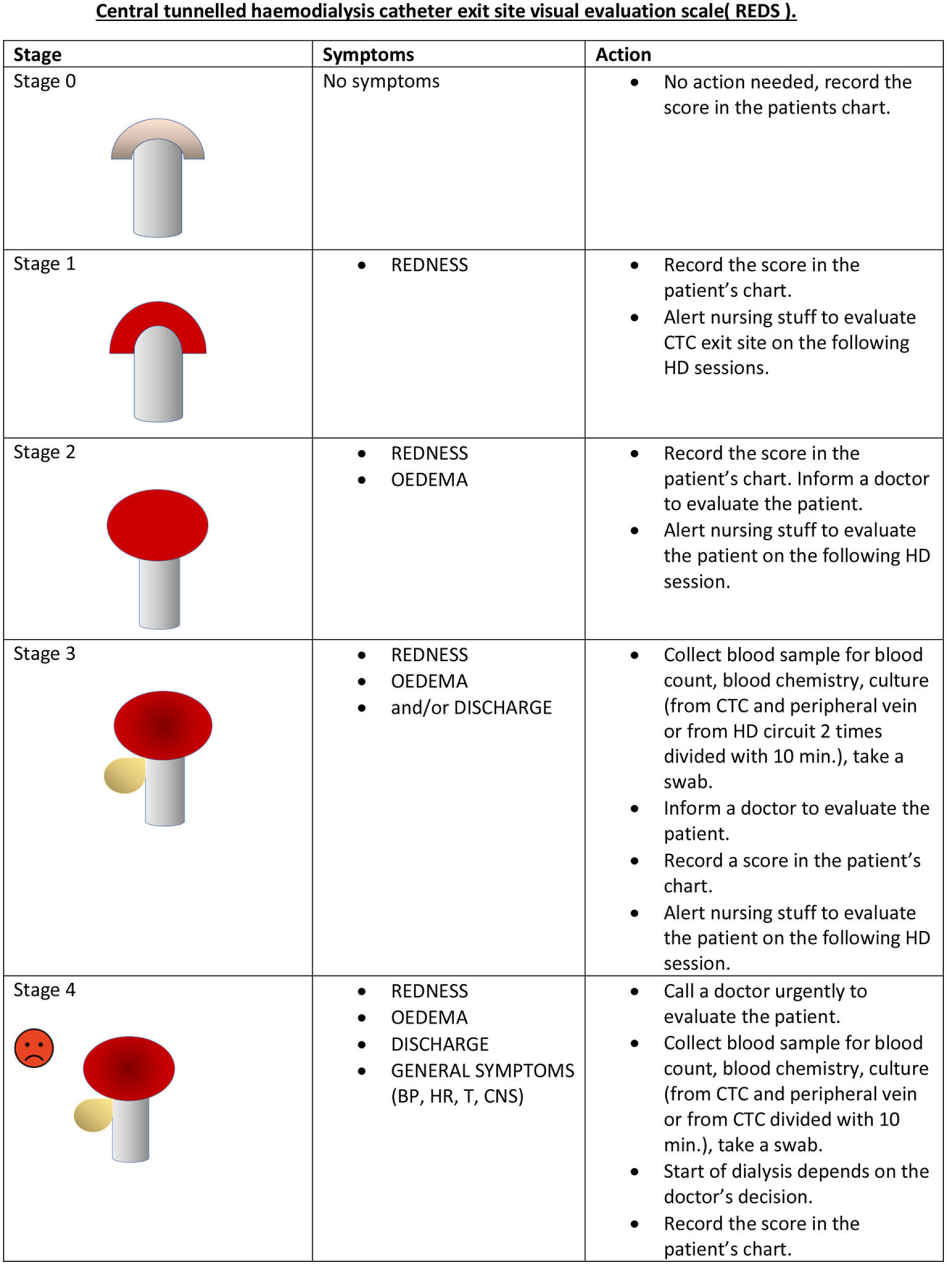
REDS scale chart (sample).

Forty-eight patients were initially included in the study. For all of them, a tunneled CTC was the only possible vascular access option. During the observation period (from 2012 until the end of 2015), five patients died due to cardiovascular events and two more due to cancer disease. One patient was transferred to peritoneal dialysis because the possibility of a further CTC use had been exhausted. After 2015, further eight death cases occurred: five—due to cardiovascular causes, one—due to complicated cholecystitis, one—due to complicated diverticulitis, and one as a consequence of kidney cancer. Finally, the study group consisted of 40 patients hemodialyzed through CTC from January 2012 until December 2015. For those individuals, complete follow-up records of a period of 2 years before and 2 years after the REDS scale application were available. The database included characteristics of the patients, REDS scoring, actions taken, and the patients' follow-up evaluation, as shown in Table 1. The REDS scale was introduced at the beginning of 2014, and its purpose was to improve observation, reporting, and management of the CTC exit site infections and related complications. The scale was based on the Twardowski scale, widely used for peritoneal dialysis catheter exit site scoring (9) and on MR VICTOR—the multiracial visual inspection catheter tool observation record, as published before (10). The REDS scale was defined as follows (Figure 1):

- 0—normal exit site (R-0, E-0, D-0, S-0).
- 1—exit site redness (R-1, E-0, D-0, S-0).
- 2—exit site redness and edema (R-1, E-1, D-0, S-0).
- 3—exit site redness and edema, and discharge or discharge only (R-1, E-1, D-1, S-0).
- 4—exit site redness and edema, tenderness and discharge, and systemic symptoms (R-1, E-1, D-1, S-1).

**Table 1:** Patients' group characteristic.

Gender *n* F/M	22/18	
Age median, range	65 years (87; 47)	
**Cause of ESKD *n* (%)**		
DM	15 (37.5%)	
ADKPD	6 (15%)	
HTN	6 (15%)	
UNKN	5 (12.5%)	
GN	4 (10%)	
NPL	4 (10%)	
**CTC site *n* (%)**	**Pre-REDS**	**REDS**
RJV	24 (60%)	20 (50%)
LJV	13 (32.5%)	14 (35%)
LSV	2 (5%)	3 (7.5%)
RSV	1 (2.5%)	3 (7.5%)
**CTC *n* (%)**	**Pre-REDS**	**REDS**
First	10 (25%)	3 (7.5%)
Second	13 (32.5%)	11 (27.5%)
Third	6 (15%)	15 (37.5%)
Fourth and further	11 (27.5%)	12 (30%)
Last option CTC *n* (%)	10 (25%)	12 (30%)

*F, females; M, males; RSV, right subclavian vein ESKD, end-stage kidney disease, DM, diabetes mellitus; ADPKD, adult polycystic disease; HTN, hypertension; UNKN, unknown; GN, glomerulonephritis; NPL, neoplasm; CTC, central tunneled catheter; RJV, right jugular vein; LJV, left jugular vein; LSV, left subclavian vein; RSV, right subclavian vein*.

Systemic symptoms were defined as body temperature >38 or <36°C, chills, hypotonia with systolic blood pressure <90 mmHg, tachycardia >90/min, and unusual patient confusion. After exit site scoring with the REDS scale, the following actions were taken accordingly:

- REDS 0—no action was taken; the score was recorded.
- REDS 1—incident was recorded in the notes, and the staff was alerted to evaluate the CTC exit site on the following HD sessions.
- REDS 2—the incident was recorded in the notes, the senior staff/doctor was informed, and the patient was evaluated. Special alertness on the following HD sessions was called for.
- REDS 3—the incident was recorded in the notes, senior staff/doctors were informed, and the patient was evaluated; blood samples were collected for tests, exit site swabs, and blood cultures, both from CTC and peripherally, were taken. An oral or intravenous antibiotic empirical treatment was initiated for 3 weeks, in accordance with our hospital infectious disease control committee guidelines (cloxacillin, amoxicillin with clavulanate, or clindamycin). The patient was evaluated using the REDS scale on every session, and the blood tests were repeated weekly. When REDS decreased to 2 and 1, the treatment was completed as planned. If the blood cultures were positive, the CTC, when possible, was removed, and a temporary catheter was inserted in a different location. When no alternative venous site was available, the CTC was exchanged for an acute line over-the-wire during antibiotic therapy, and then modified in relevance with the microbiologic tests results.
- REDS 4—the senior staff and the doctor were informed immediately; the exit site swab and blood samples were taken for cultures from the CTC and peripheral vein, as well as for blood tests. Empirical antibiotic was given (mostly vancomycin alone or with ceftazidime or with gentamycin). Afterwards, the catheter was removed when possible, and a temporary catheter was inserted in a different location. When no alternative sites could be found, the CTC was exchanged for an acute line over-the-wire. In case of an indication for urgent hemodialysis (pulmonary edema, life-threatening hyperkalemia), the procedure was carried out through the infected CTC, and further actions were taken thereafter.

Tunnel infection (TI) was diagnosed when pain, swelling, redness of skin along the CTC, as well as purulent discharge from the exit site were observed. The same actions were taken as in the REDS 4 score. CRBSI was suspected when systemic symptoms were present, confirmed by blood and culture tests, with or without REDS 3 to 4 or tunnel infection signs. However, in the case of the last option CTC, the patient was put on systemic empirical antibiotic, with blood cultures taken from the line and peripherally, at the beginning of the evaluation and 48 h later. In case of no response to treatment, the CTC was exchanged over-the-wire for a temporary line. The next exchange of the catheter was carried out after clinical improvement, with negative blood culture tests, and the regression of the inflammation confirmed by the blood test. The nurses, as well as senior medical staff, were trained in the use of the REDS scale; appropriate materials were prepared, including posters and forms, which were attached to the patients' records. It was crucial, as the nursing staff was responsible for the CTC dressing change, exit site scoring and, when necessary, the sample collection. On the basis of that initial evaluation, the doctor was informed to take proper action. The results were analyzed monthly, and a complete report was prepared yearly from January 2014 until December 2018.

In pre-REDS period, the exit site infection was defined, at the nursing staff's discretion, by the presence of redness, swelling, and discharge. In that case, blood samples and skin swabs were taken routinely and an empirical antibiotic was given. When discharge was absent, blood was tested and the beginning of treatment depended on the clinician's decision, i.e., when CRP and WBC were significantly elevated. The presence of discharge without the accompanying exit site skin changes was an indication for a swab, blood test, and empirical treatment based on the obtained results, and modified accordingly to microbiologic report. In the case of the appearance of systemic symptoms like fever, chills, hypotonia, tachycardia, and unusual confusion suggesting CRBSI—after other sources of infection were excluded (i.e., respiratory tract, urinary tract, etc.)—blood samples for culture and tests were obtained peripherally and from CTC, followed by empiric antibiotic administration, as mentioned above.

All investigated procedures aimed at the treatment of complications threatening the vascular access for life-supporting hemodialysis. The CTC exit site REDS scale scoring was an improvement of the already existing vital signs recording and physical examination performed before every hemodialysis session. Due to this fact, an opinion from the Opole University Bioethics Committee was expressed that this type of observational study dictated by the clinical needs does not require authorization. Informed consent was obtained from all the individual patients before the procedure requiring catheter removal and reimplantation. Those procedures were conducted in accordance with the ethical standards of the Declaration of Helsinki from 1964 and its later amendments.

Nominal variables are presented as *n* (% of total) and continuous variables as mean (SD). All the CTC-related infectious complication were presented as episode per 1,000 catheter-days (/1,000 cd), then a cumulative incidence of all episodes was calculated using the Kaplan-Meier survival analysis method, including 95% confidence interval. Log-rank Cox-Mantel test was used to compare cumulative incidence level between pre-REDS and REDS periods (based on 4-year data). Additionally, the Cox hazard ratio regression analysis models were used to identify the factors significantly impacting the incidence of infections. The analyses were carried out with the use of statistical software R (version 3.5.2; http://cran.r-project.org).

## Results

Patient group characteristics are presented in Table 1. In 40 patients' population, there were more women (22 vs. 18 men, respectively), medium aged of 65 years (87; 47), with diabetes mellitus (37.5%) as dominating cause of ESKD and dialyzed through a CTC mostly inserted into the right internal jugular vein (62.5%). At the beginning of observation, in 10 (25%) patients, it was the only first CTC, in 11 (27.5%) the fourth one and more, of particular note—in 10 (25%) it was already the last option of the CTC site. When REDS was introduced, three (7.5%) patients still had their first CTC, 11 (27.5%) had a second line, and in 12 (30%) the CTC was inserted into the last available central vein site.

**Figure 2:**
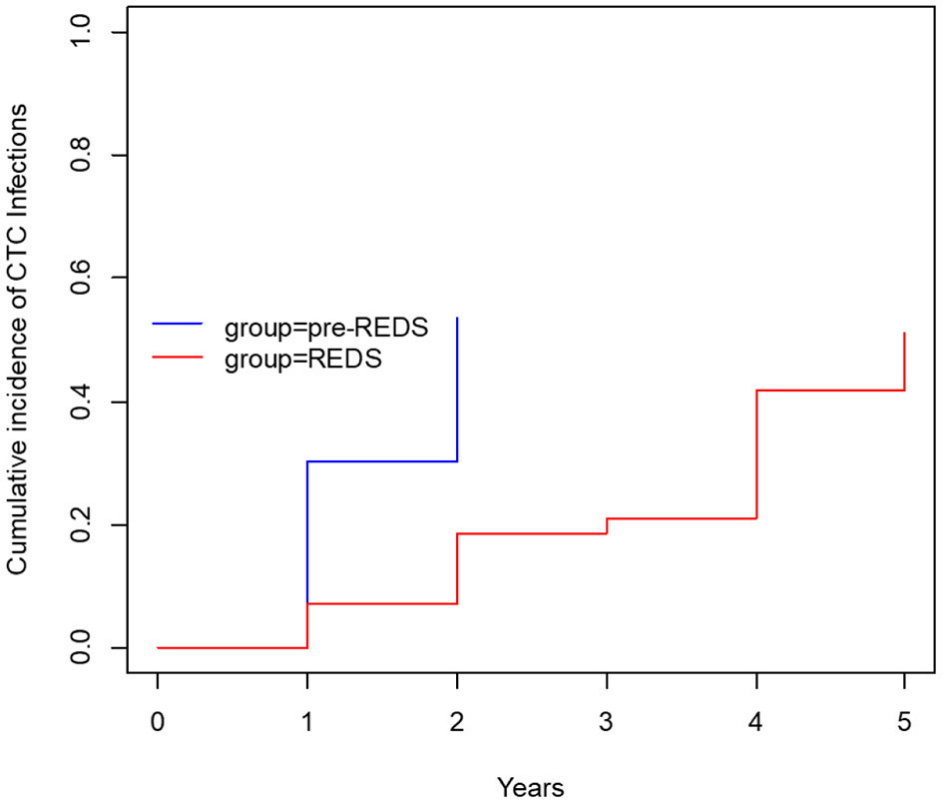
The cumulative incidence of ESI (before and after REDS).

**Figure 3:**
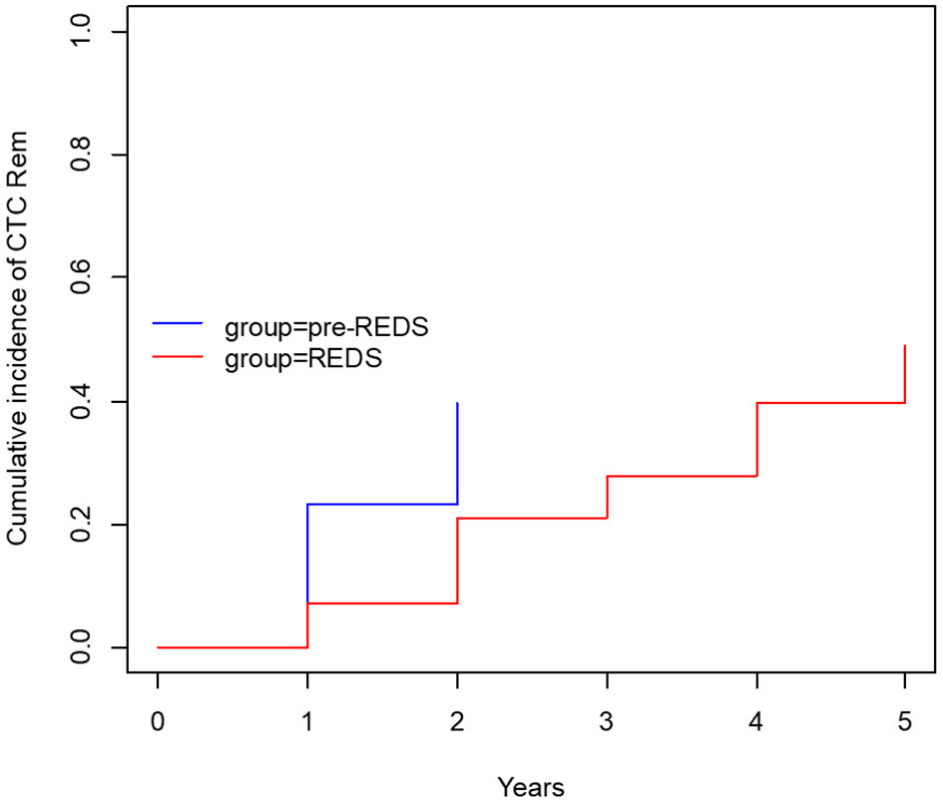
The cumulative incidence of CTC removal with concurrent ESI (R3 and R4) and CRBSI (before and after REDS).

After the REDS scale introduction, ESI (calculated sum of R3 and R4 incidence episodes) occurrence decreased to the rate of 0.26 and 0.47 episode/1,000 cd in 2014 and 2015, respectively, compared with 0.89 episode/1,000 cd in both 2012 and 2013, in pre-REDS time (Table 2). From 2014 until the end of 2015, the R3 stage was recorded at a rate between 0.2 and 0.27/1,000 cd; in parallel, R4 appeared with incidence 0.06 and 0.2/1,000 cd. All cases of REDS 2 were successfully treated with antibiotics (10 cases and 8 cases in 2014 and 2015, respectively), and no patient required CTC removal. There were three and four cases of REDS 3 stage diagnosed in 2014 and 2015, respectively, and in one (in 2014) and two cases (in 2015), antibiotic treatment was successful. All patients with REDS 4 required line excision. Tunnel infection occurrence also decreased, from the range of rates 0.47 to 0.34/1,000 cd in pre-REDS period to the range between 0.13 and 0.27/1,000 cd in the first 2 years of REDS period. Fewer CTCs were also removed due to infections (0.7 episode/1,000 cd vs. 0.4/1,000 cd; pre-REDS vs. REDS time, respectively). Three (0.2/1,000 cd) cases of IE, one (0.07/1,000 cd) case of OMS, and five (0.33/1,000 cd) cases of DSC were recorded in pre-REDS in comparison with one case of DSC (0.07/1,000 cd) and one case of IE in REDS application period; however, there was only one fatal CTC infection-related episode during the study time. The results were further analyzed with the Log-rank Cox-Mantel test, which showed a cumulative ESI incidence of 53.5%, 95% CI [35.9%; 66.2%] in pre-REDS time and 18.6%, 95% CI [6.1%; 29.4%] in REDS time, and the difference was statistically significant (log-rank *p* < 0.001; Table 3; Figure 2). There were also significantly fewer episodes of ESI complicated with CRBSI requiring CTC removal (18.6%, 95% CI [6.1%; 29.4%] vs. 4.7%, 95% CI [0.0; 10.7%]; log-rank *p* = 0.04, in pre-REDS and REDS time, respectively; Table 3, Figure 3). For CTC removal, the difference before and after REDS was of marginal significance, with cumulative incidence greater in pre-REDS time (*p* = 0.05; Table 3). However, for TI and CRBSI with no pre-existing ESI episode, no significant differences in cumulative incidence before and after REDS were confirmed. An additional analysis, using the Cox hazard ratio model, revealed no significant relationship between the occurrence of ESI, TI, CRBSI, or CTC removal and the age, gender, ESKD, or CTC site (Supplementary Tables 1–4). The REDS scale use did not influence significantly the incidence of the CTC thrombotic dysfunction (0.57/1,000 cd; 23.3%, 95% CI [9.5–34.9%] vs. 0.51/1,000 cd; 16.3%, 95% CI [4.5–26.6%]; log-rank *p* = 0.5).

**Table 2:** Incidence of CTC-related infectious complications in pre-REDS and REDS period of time.

	**Pre-REDS**		**REDS**				
	**2012**	**2013**	**2014**	**2015**	**2016**	**2017**	**2018**
Patients (*n*)	40	40	40	40	38	33	32
ESI	0.89	0.89	0.26	0.47	0.23	0.47	0.64
R3	N/A	N/A	0.2	0.27	0.13	0.27	0.54
R4	N/A	N/A	0.06	0.2	0.1	0.1	0.2
TI	0.47	0.34	0.13	0.27	0.06	0.13	0.2
CRBSI	0.54	0.61	0.27	0.41	0.27	0.34	0.34
CTC removal	0.68	0.61	0.2	0.41	0.47	0.61	0.75
IE (*n*)	0.13 (2)	0.13 (1)	0	0.07 (1)	0	0.08 (1)	0
OMS % (*n*)	0.07 (1)	0	0	0	0	0	0
DSC % (*n*)	0.13 (2)	0.2 (3)	0.07 (1)	0	0.07 (1)	0	0.09 (1)

*All data presented as rate per 1,000 catheter days or number of episodes in brackets. ESI, exit site infection (in REDS time R3 and R4 in total and separately); R3, REDS stage 3; R4, REDS stage 4; TI, tunnel infection; CRBSI, catheter-related blood stream infection; CTC rem., central tunneled catheter removal; IE, infective endocarditis; OMS, osteomyelitis; DSC, discitis*.

**Table 3:** Comparison of cumulative incidence of CTC-related infectious complications in pre-REDS and REDS period of time.

	**P-REDS (2012–2013)**		**REDS (2014–2015)**		***p* (log-rank)**
	**2-year Cum. incidence**	**CI 95%**	**2-year Cum. incidence**	**CI 95%**	
**ESI**	**53.5%**	**35.9–66.2%**	**18.6%**	**6.1–29.4%**	**<0.001**
TI	25.6%	11.3–37.5%	14.0%	2.9–23.7%	0.200
CRBSI	34.9%	19.0–47.7%	23.3%	9.5–34.9%	0.200
**CTC rem**.	**39.5%**	**23.0–52.5%**	**20.9%**	**7.8–32.2%**	**0.050**
**ESI** **+** **CRBSI** **+** **CTC rem**.	**18.6%**	**6.1–29.4%**	**4.7%**	**0.0–10.7%**	**0.040**

*ESI, exit site infection (in REDS R3 and R4 in total); TI, tunnel infection; CRBSI, catheter-related blood stream infection; CTC, central tunneled catheter; CTC rem., CTC removal*.

*Significant differences in presented values were highlighted*.

## Discussion

Infections are the second leading cause of death among dialysis patients, and central venous catheter (CVC) use for hemodialysis (HD) is associated with the increased rates of infection, as compared with other types of vascular access (1–3). For that reason, careful monitoring of the tunneled catheters as a vascular access is the supreme task of the dialysis staff, which should diminish mortality and reduce hospital costs (8, 11–13). Presented results demonstrate that the formal protocol of exit site evaluation using the introduced REDS scale significantly decreases the frequency of exit site infections, and particularly the exit site infections accompanied by CRBSI, as compared with the period of 2 years in which that evaluation had been left to nurses' discretion. All the cases of early detected mild infection (REDS 2) that was properly treated allowed to avoid catheter removal (10 and 8 cases in 2014 and 2015, respectively). Among patients with advanced stage of infection (REDS 3), only few CTC survived (one per three cases and two per four cases in 2014 and 2015, respectively). ESI followed by CRBSI occurred with a frequency of 18.6% before REDS introduction and in 4.7% after that. It is noteworthy that the rate of these two kinds of infections even in the earlier period before REDS implementation remained in the lower limits reported in literature (1–4). The reduction of the ESI and CRBSI exerted positive impact on the frequency of the CTC removal, which dropped from 39.5 to 20.9% (*p* = 0.05; Table 3; Figure 3). REDS did not affect significantly the rate of CRBSI not associated with the ESI infection, but significantly less catheters were removed because of CRBSI. In addition, the incidence of the most severe cases of CRBSI complicated by infective endocarditis and metastatic infection declined from 12.5 to 2.5%. It is also lower than the numbers reported in the literature (1–7). The overall occurrence of CRBSI in the study group was <0.9/1,000 patient catheter days before REDS applications and subsequently decreased to 0.6 episode. It is below the targets recommended by the guidelines (4, 5, 12, 13). Respecting the limitations of this study implied by the small sample size and its retrospective nature, the presented observations strongly indicate that scrutiny in the exit site estimation by nursing staff will always remain of utmost importance, irrespective of progress in cuff technology. The REDS scale appears to be a useful tool for fulfilling this task.

## Conclusions

The introduction of the formal evaluation of the tunneled catheter exit site before each dialysis session using the innovative REDS scale reduced significantly the frequency of the exit site infections and the associated catheter-related bloodstream infections in the long-term catheter carriers.

## Data Availability Statement

The original contributions presented in the study are included in the article/Supplementary Material, further inquiries can be directed to the corresponding author.

## Ethics Statement

Ethical review and approval was not required for the study on human participants in accordance with the local legislation and institutional requirements. The patients/participants provided their written informed consent to participate in this study.

## Author Contributions

TP was an author of REDS scale, leading Interventional Nephrologist in the centre performing CTC insertion procedure and was responsible for study concept, data collection, analysis and manuscript preparation. ES was a Senior Renal Nurse leading dialysis unit nursing staff and was responsible for REDS scale introduction, quality monitoring and data collection. MK was the Department Director and was responsible for study concept, data analysis and manuscript preparation. All authors contributed to the article and approved the submitted version.

## Conflict of Interest

The authors declare that the research was conducted in the absence of any commercial or financial relationships that could be construed as a potential conflict of interest.
