# Supplementary material for: The Impact of the Introduction of Innovative REDS Scale for the Evaluation of Central Tunnelled Catheter (CTC) Exit Site on Infection Prevention in Long-Term Haemodialyzed Patients
Source: Front Surg. 2021 Apr 9;8:629367. doi: 10.3389/fsurg.2021.629367 (PMC8062969; doi:10.3389/fsurg.2021.629367)
Supplement: Supplementary file 1 [file Data_Sheet_1.pdf]

Supplementary materials.

Table S 1. Multivariate regression analysis of ESI incidence variables with Cox hazard ratio method

Variable	Coefficient	SE (coefficient)	*P*	Exp (coefficient)
Age, years	-0.02	0.04	0.571	0.98
Sex, male	0.21	0.60	0.720	1.24
ESKD, DM	-0.59	0.75	0.437	0.57
ESKD, GN	-0.45	0.76	0.559	0.64
ESKD, HTN	-1.65	1.19	0.167	0.19
ESKD, NPL	-0.72	1.01	0.477	0.49
ESKD, UNKN	-1.56	1.20	0.196	0.21
CTC site, LJV	-0.16	71.5	0.998	0.0001
CTC site, RJV	0.10	0.54	0.860	1.10
CTC site, LSV	-0.20	1.3	0.291	0.17
CTC site, RSV	-0.16	71.5	0.998	0.0001

ESKD – end-stage kidney disease, DM - diabetes mellitus, ADPKD - adult polycystic disease, HTN - hypertension, UNKN - unknown, GN - glomerulonephritis, NPL - neoplasm, CTC - central tunnelled catheter, RJV - right jugular vein, LJV - left jugular vein, LSV - left subclavian vein, RSV - right subclavian vein, RSV - right subclavian vein.

Table S 2. Multivariate regression analysis for TI incidence variables with Cox hazard ratio method

Variable	Coefficient	SE (coefficient)	*P*	Exp (coefficient)
Age, years	0.06	0.04	0.174	1.06
Sex, male	-0.20	0.67	0.764	0.81
ESKD, DM	-1.71	1.07	0.109	0.18
ESKD, GN	0.41	0.88	0.638	1.51
ESKD, HTN	-2.06	1.39	0.142	0.13
ESKD, NPL	-1.67	1.33	0.209	0.19
ESKD, UNKN	-1.14	1.10	0.299	0.32
CTC site, LJV	-0.16	88.70	0.999	0.0001
CTC site, RJV	-0.18	72.0	0.734	0.34
CTC site, LSV	0.13	6.28	0.836	1.14
CTC site, RSV	-0.17	88.70	0.998	0.0001

ESKD – end-stage kidney disease, DM - diabetes mellitus, ADPKD - adult polycystic disease, HTN - hypertension, UNKN - unknown, GN - glomerulonephritis, NPL - neoplasm, CTC - central tunnelled catheter, RJV - right jugular vein, LJV - left jugular vein, LSV - left subclavian vein, RSV - right subclavian vein, RSV - right subclavian vein.

Table S 3. Multivariate regression analysis for CRBSI incidence variables with Cox hazard ratio method

Variable	Coefficient	SE (coefficient)	*P*	Exp (coefficient)
Age, years	-0.03	0.03	0.216	0.97
Sex, male	-0.07	0.48	0.883	0.93
ESKD, DM	0.73	0.68	0.281	2.08
ESKD, GN	-0.05	0.78	0.953	0.95
ESKD, HTN	0.23	0.85	0.777	1.27
ESKD, NPL	0.75	0.88	0.394	2.12
ESKD, UNKN	1.26	0.74	0.090	3.51
CTC site, LJV	-0.30	0.41	0.469	0.74
CTC site, RJV	0.45	0.65	0.54	0.83
CTC site, LSV	-0.17	54.4	0.998	0.0001
CTC site, RSV	-0.18	54.4	0.997	0.0001

ESKD – end-stage kidney disease, DM - diabetes mellitus, ADPKD - adult polycystic disease, HTN - hypertension, UNKN - unknown, GN - glomerulonephritis, NPL - neoplasm, CTC - central tunnelled catheter, RJV - right jugular vein, LJV - left jugular vein, LSV - left subclavian vein, RSV - right subclavian vein, RSV - right subclavian vein.

Table S 4. Multivariate regression analysis for CTC incidence variables removal with Cox hazard ratio method

Variable	Coefficient	SE (coefficient)	*P*	Exp (coefficient)
Age, years	0.02	0.03	0.505	1.02
Sex, male	0.05	0.46	0.909	1.05
ESKD, DM	-0.47	0.69	0.497	0.63
ESKD, GN	0.32	0.66	0.629	1.37
ESKD, HTN	-0.84	0.85	0.320	0.43
ESKD, NPL	-0.87	0.95	0.360	0.42
ESKD, UNKN	-0.01	0.74	0.994	0.99
CTC site, LJV	0.34	0.76	0.977	0.81
CTC site, RJV	-0.17	53.9	0.998	0.0001
CTC site, LSV	-0.14	0.43	0.737	0.87
CTC site, RSV	-0.18	53.9	0.997	0.0001

ESKD – end-stage kidney disease, DM - diabetes mellitus, ADPKD - adult polycystic disease, HTN - hypertension, UNKN - unknown, GN - glomerulonephritis, NPL - neoplasm, CTC - central tunnelled catheter, RJV - right jugular vein, LJV - left jugular vein, LSV - left subclavian vein, RSV - right subclavian vein, RSV - right subclavian vein.
